# Tonsil-Derived Mesenchymal Stem Cells Differentiate into a Schwann Cell Phenotype and Promote Peripheral Nerve Regeneration

**DOI:** 10.3390/ijms17111867

**Published:** 2016-11-09

**Authors:** Namhee Jung, Saeyoung Park, Yoonyoung Choi, Joo-Won Park, Young Bin Hong, Hyun Ho Choi Park, Yeonsil Yu, Geon Kwak, Han Su Kim, Kyung-Ha Ryu, Jae Kwang Kim, Inho Jo, Byung-Ok Choi, Sung-Chul Jung

**Affiliations:** 1 Department of Biochemistry, School of Medicine, Ewha Womans University, Seoul 07985, Korea; polypola@ewhain.net (N.J.); saeyoung@ewha.ac.kr (S.P.); yychoi27@ewhain.net (Y.C.); joowon.park@ewha.ac.kr (J.-W.P.); 2 Stem Cell & Regenerative Medicine Institute, Samsung Medical Center, Seoul 06351, Korea; ybhong@skku.edu; 3 Department of Neuroscience and Behavioral Biology, Emory University, Atlanta, GA 30322, USA; hcpark2@emory.edu; 4 Department of Molecular Medicine, School of Medicine, Ewha Womans University, Seoul 07985, Korea; rys75@ewha.ac.kr (Y.Y.); inhojo@ewha.ac.kr (I.J.); 5 Department of Health Sciences and Technology, SAIHST, Sungkyunkwan University, Seoul 06351, Korea; kgun903@naver.com; 6 Department of Otorhinolaryngology-Head & Neck Surgery, School of Medicine, Ewha Womans University, Seoul 07985, Korea; sevent@ewha.ac.kr; 7 Department of Pediatrics, School of Medicine, Ewha Womans University, Seoul 07985, Korea; ykh@ewha.ac.kr; 8 Department of Orthopedic Surgery, School of Medicine, Ewha Womans University, Seoul 07985, Korea; kimjk@ewha.ac.kr; 9 Department of Neurology, Samsung Medical Center, Sungkyunkwan University School of Medicine, Seoul 06351, Korea; bochoi77@hanmail.net

**Keywords:** tonsil-derived mesenchymal stem cells, Schwann cell, differentiation, peripheral nerve, regeneration

## Abstract

Schwann cells (SCs), which produce neurotropic factors and adhesive molecules, have been reported previously to contribute to structural support and guidance during axonal regeneration; therefore, they are potentially a crucial target in the restoration of injured nervous tissues. Autologous SC transplantation has been performed and has shown promising clinical results for treating nerve injuries and donor site morbidity, and insufficient production of the cells have been considered as a major issue. Here, we performed differentiation of tonsil-derived mesenchymal stem cells (T-MSCs) into SC-like cells (T-MSC-SCs), to evaluate T-MSC-SCs as an alternative to SCs. Using SC markers such as *CAD19*, *GFAP*, *MBP*, *NGFR*, *S100B*, and *KROX20* during quantitative real-time PCR we detected the upregulation of *NGFR*, *S100B*, and *KROX20* and the downregulation of *CAD19* and *MBP* at the fully differentiated stage. Furthermore, we found myelination of axons when differentiated SCs were cocultured with mouse dorsal root ganglion neurons. The application of T-MSC-SCs to a mouse model of sciatic nerve injury produced marked improvements in gait and promoted regeneration of damaged nerves. Thus, the transplantation of human T-MSCs might be suitable for assisting in peripheral nerve regeneration.

## 1. Introduction

Schwann cells (SCs) are the glial cells of peripheral nerves that wrap around axons to form myelin in the peripheral nervous system. The development of SCs involves a series of steps in which neural crest cells give rise to SC precursors and then differentiate into immature SCs. These finally differentiate into mature SCs that include both myelinating and nonmyelinating types [1].

SCs are indispensable mediators of repair after nervous tissue injury [2]. They are also involved in the pathogenesis of many diseases, including genetic disorders such as Charcot–Marie–Tooth disease, hereditary neuropathy with liability to pressure palsies, and metabolic diseases such as diabetic neuropathy [3,4,5]. SCs have been proposed as a potential cell source for transplantation for functional peripheral nerve recovery. Endogenous SCs produce various neurotrophic factors, cytokines, and extracellular matrix molecules, thereby providing structural support and guidance for regenerating axons [6]. Autologous SC transplantation has shown promising clinical results, such as in remyelinating damaged axons and restoring their electrical conduction properties [7]. However, to obtain SCs from nerve biopsies, another functional nerve must be sacrificed for the in vitro expansion of the desired cell line. Furthermore, because there are technical difficulties in culturing SCs, securing sufficient numbers is not easy. Therefore, it would be desirable to harvest other cell sources with extensive self-renewal capacity, broad differentiation potential, and readily accessible properties.

Mesenchymal stem cells (MSCs) are multipotent stem cells derived from various tissues, which have the capacity to differentiate into several mesodermal lineages, including osteoblasts, chondroblasts, and adipocytes [8]. These diverse multipotent MSCs can also be differentiated into cells of the ectodermal lineage including neurons and glial cells. Various studies using skin-derived MSCs, adipose tissue-derived MSCs (AMSCs), bone marrow-derived MSCs, and umbilical cord-derived MSCs have been performed in attempts to differentiate them into SCs under diverse conditions [9,10,11,12]. Although bone marrow-derived MSCs are one of the most intensively studied types of MSC associated with SC differentiation, they are limited in terms of clinical applications because of their low yields and the need for invasive procedures to isolate them [13]. Moreover, their proliferation rates and differentiation potentials have been shown to decrease with donor age.

Tonsil-derived MSCs (T-MSCs), isolated from palatine tonsils as “waste” tissues during tonsillectomy, have been reported recently as a new class of MSC [14,15]. Human tonsils are lymphoepithelial tissues that act as immune organs until puberty, and undergo atrophy during aging. As with other MSCs, T-MSCs also exhibit self-renewal capacity, multilineage differentiation properties, and immunosuppressive characteristics [15]. In particular, the high proliferation rate of T-MSCs is very important for quantitative recovery and for the establishment of dependable cell lines. Several studies have confirmed that T-MSCs express typical MSC cell surface markers and can differentiate into mesodermal lineages [14,15,16].

Here, we demonstrate that T-MSCs can differentiate into SCs (T-MSC-SCs). To evaluate this, quantitative reverse transcription polymerase chain reaction (RT–qPCR), Western blotting, and immunostaining were performed. The T-MSC-SCs were cocultured with mouse dorsal root ganglion (DRG) neurons to investigate the formation of myelin sheaths on axons, and T-MSC-SCs were transplanted into mice carrying a sciatic nerve injury. To evaluate the secretion of neurotrophic factors, neurite outgrowths of NSC34 mouse motor neurons were measured when they were cultured with conditioned medium (CM) obtained from T-MSC-SC cultures. We hypothesized that T-MSC-SCs might serve as an alternative cell source for native SCs and could be used for autologous transplantation therapy in cases of peripheral nerve injury.

## 2. Results

### 2.1. Differentiated Tonsil-Derived Mesenchymal Stem Cells (T-MSCs) Exhibit Schwann Cell (SC)-Like Morphology

**Figure 1:**
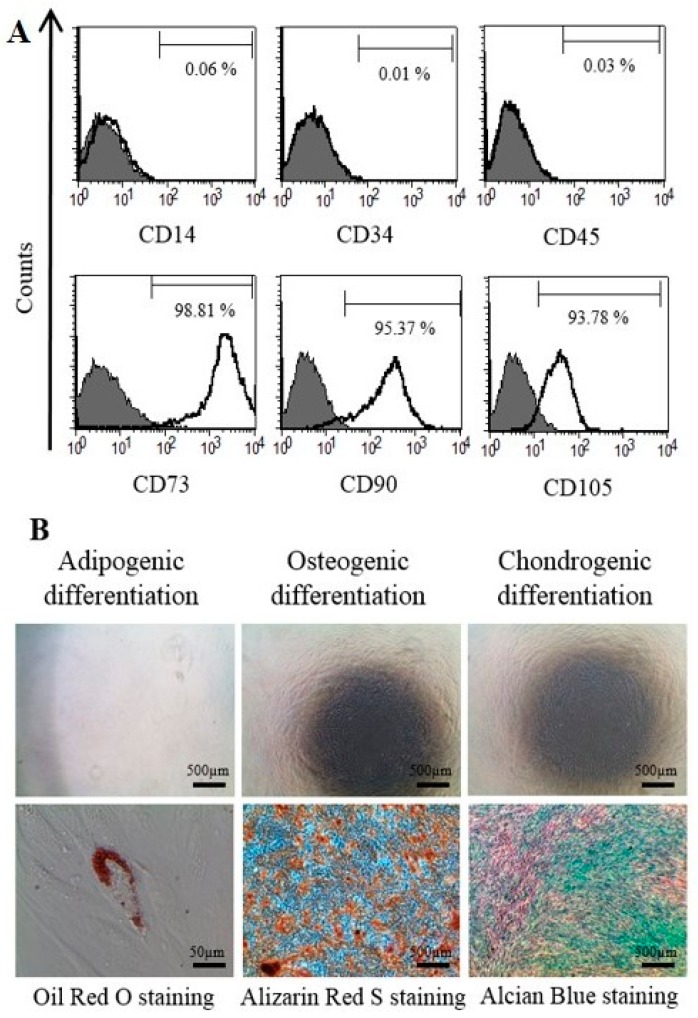
Characterization of tonsil-derived mesenchymal stem cells (T-MSCs) as mesenchymal stem cells: (**A**) the patterns of mesenchymal stem cells (MSC) surface markers were determined fluorescence-activated cell sorter (FACS) Calibur flow cytometer. Gray histogram profile indicates the isotype control, and white histogram indicates the specific antibody; and (**B**) T-MSCs differentiated into adipocytes, osteoblasts and chondrocytes, as determined by Oil Red O, Alizarin Red S staining, and Alcian blue, respectively. Negative control staining (**upper row**) and mesodermal differentiated T-MSC (**lower row**). Scale bars indicate each length.

**Figure 2:**
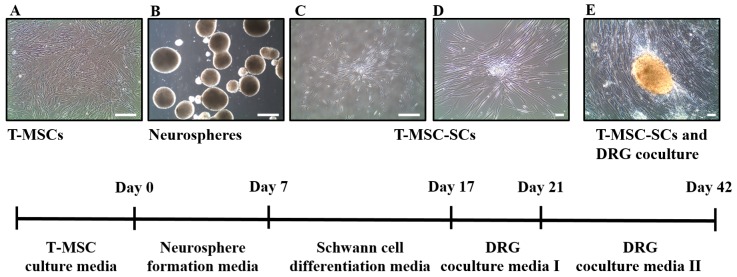
Schematic diagram for the differentiation of T-MSCs into T-MSC-SCs. The method for differentiation of T-MSCs into T-MSC-SCs comprised several steps, including: (**A**) T-MSC expansion; (**B**) neurosphere formation; (**C**,**D**) SC differentiation; and (**E**) dorsal root ganglion (DRG) coculture. Images were acquired on an Olympus IX51 microscope equipped with NA0.30 objective lenses (original magnification in **A**, **B**, and **C**, ×40; in **D**, and **E**, ×100). Scale bar = 50 µm.

To examine cell surface markers and mesodermal differentiation capacity of T-MSCs, flow cytometry analyses and mesodermal differentiation were determined, as described [17]. Figure 1A shows the pattern of staining of the MSC surface markers. T-MSCs exhibited a lack of expression of the CD14, CD34, and CD45 surface markers and expressed the CD73, CD90 and CD105 cell surface markers. We also investigated the capacity of T-MSCs to differentiate into mesodermal cells using a differentiation culture medium. Adipogenic differentiation was confirmed by Oil Red O staining, osteogenic differentiation was confirmed by Alizarin Red S staining, and chondrogenic differentiation was confirmed by Alcian blue staining (Figure 1B). Human T-MSCs recovered within 5–8 passages after initial plating of the primary culture and appeared to be monolayers of large flat cells. They were replated into plastic dishes containing DMEM/F12 medium supplemented with EGF, bFGF, and B27. Many spheres of floating cells appeared after seven days in this medium. These neurospheres were triturated and replated into laminin-coated dishes with SC differentiation medium (Figure 2). The mechanically dissociated cells showed morphological changes such as elongated bipolar or tripolar spindle shapes after 10 days of differentiation (Figure 2C,D). The T-MSC-SCs also displayed thinner cytoplasmic extensions and larger nuclei than did the undifferentiated T-MSCs.

### 2.2. RT–qPCR

**Figure 3:**
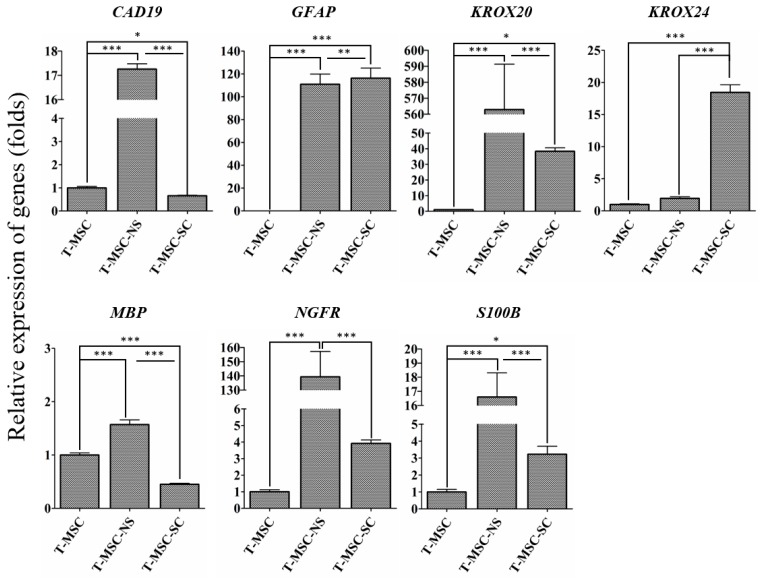
RT-qPCR analyses of *CAD19*, *GFAP*, *KROX20*, *KROX24*, *MBP*, *NGFR*, and *S100B* genes in T-MSCs, T-MSC-NSs and T-MSC-SCs. Expression levels were normalized against expression of the housekeeping gene encoding glyceraldehyde 3-phosphate dehydrogenase (GAPDH), and the results are reported as ratios of the marker gene expression versus undifferentiated T-MSCs. The calculation of relative gene expression level was analyzed using the comparative Ct method ($2^{-\Delta \Delta C_{t}}$). Data are presented as the mean ± SE of at least three experiments. Statistical analysis used one-way ANOVA followed by Newman–Keuls multiple comparison tests (* *p* < 0.05; ** *p* < 0.01; *** *p* < 0.001).

To determine the differentiation potential of SC-like cells, we examined the expression of cadherin 19 (CAD19) as a SC precursor marker, and glial fibrillary acidic protein (GFAP), nerve growth factor receptor (NGFR), and S100 calcium binding protein B (S100B) as SC markers (Figure 3). Each marker for SCs can characterize each stage of the lineage. GFAP, NGFR, and S100B are typical immature SC markers. Therefore, they are absent or present at very low levels in precursors and neural crest cells, but are expressed in immature SCs. Cad19 is mainly expressed in precursor stages in the rat; however, chicken Cad19 is not expressed in SCs until late stages of development. Mature myelinating cells are characterized by upregulation of the transcription factor Krox20 and myelin-related proteins such as myelin basic protein (Mbp) [18,19,20,21]. With the exception of *KROX24*, all of the SC markers were highly expressed at the neurosphere stage. After SC differentiation, the expression levels of *GFAP*, *NGFR*, *S100B*, *KROX20*, and *KROX24* increased, whereas the levels of *MBP* and *CAD19* decreased. The expression level of *MBP*, a specific marker of the mature stage of SC development, decreased at the stage of SC differentiation. These data confirmed the potential of human T-MSCs to differentiate into SCs.

### 2.3. Expression of Nerve Growth Factor Receptor (NGFR) and Glial Fibrillary Acidic Protein (GFAP) Proteins Confirmed by Immunofluorescence and Western Blotting

**Figure 4:**
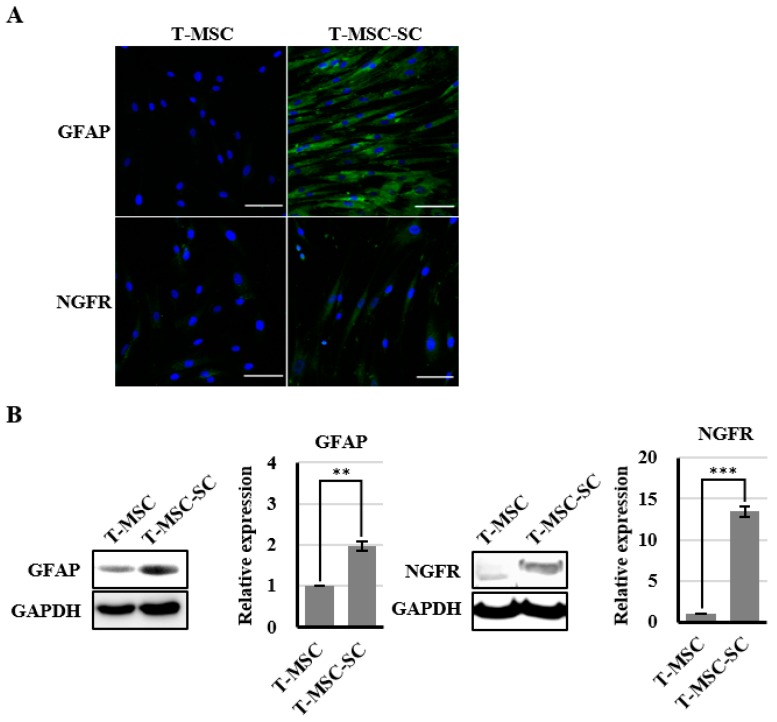

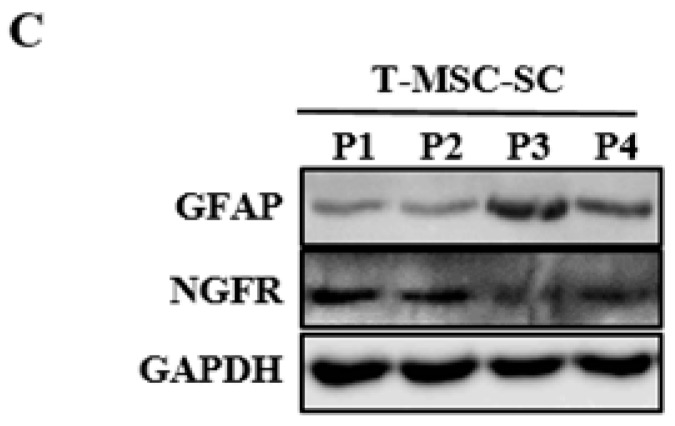
Identification of SC markers in T-MSCs and T-MSC-SCs: (**A**) immunocytochemical staining for GFAP (blue, DAPI; green, GFAP) and NGFR (blue, DAPI; green, NGFR) expression levels were compared before and after SC induction; (**B**) Western blot and quantitation graphs of GFAP and NGFR expression levels were compared between T-MSC and T-MSC-SC cells; and (**C**) GFAP and NGFR expressions in T-MSC-SCs were sustained over additional passages. The constitutively expressed GAPDH protein was used as a positive loading control. Data are presented as the mean ± SE of at least three experiments. The statistical analysis was performed using Student’s *t*-test (** *p* < 0.01; *** *p* < 0.001). Scale bar = 100 µm.

To determine the molecular features of T-MSC-SCs, immunocytochemistry and Western blotting using antibodies against NGFR and GFAP were performed both before and after SC differentiation (Figure 4). The NGFR protein was undetectable before differentiation but was strongly detectable by immunofluorescence staining and Western blotting after differentiation. The ratio of NGFR-positive cells was 67.6% ± 17.4%. Similar to NGFR, nearly all cells also expressed GFAP after SC differentiation. However, GFAP proteins were also detected in undifferentiated T-MSCs by Western blotting. During an additional three passages, the expression levels of GFAP and NGFR proteins were well sustained.

### 2.4. Conditioned Medium (CM) from SC-Like Cells Differentiated from Tonsil-Derived Mesenchymal Stem Cell (T-MSC-SCs) Promoted Neurite Outgrowth of NSC34 Motor Neurons

**Figure 5:**
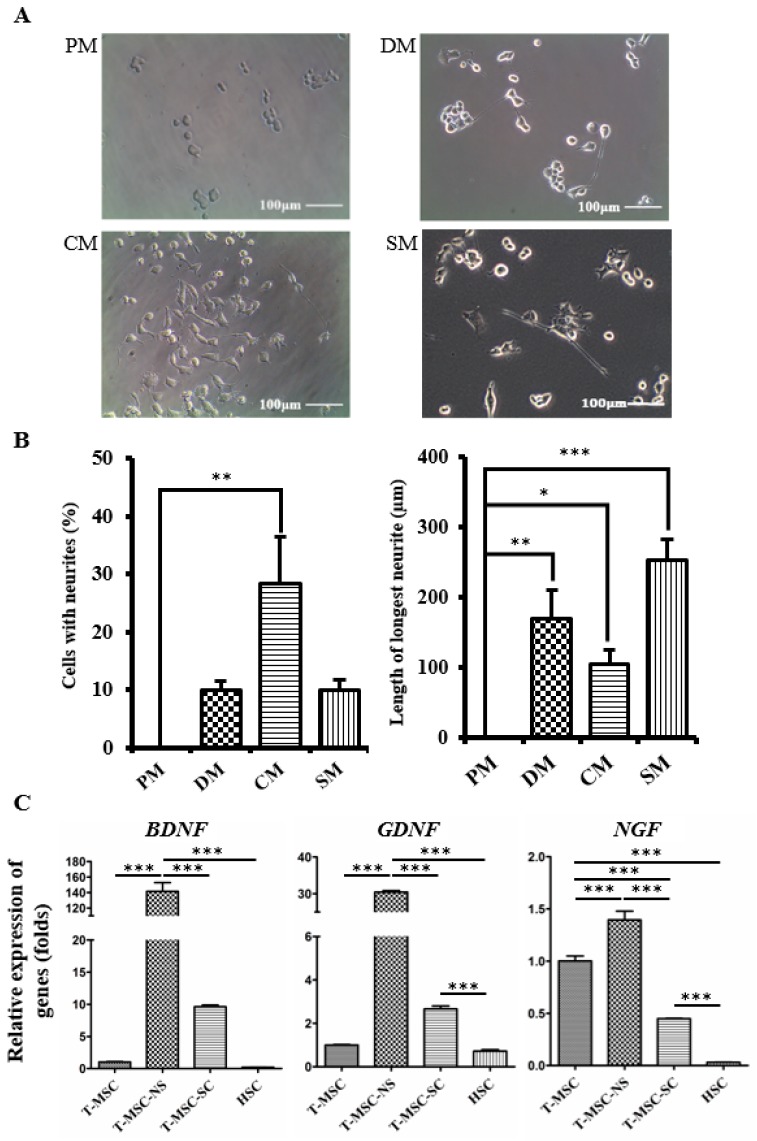
T-MSC-SCs promote neurite outgrowth in NSC34 cells: (**A**) NSC34 cells were grown in PM, DM, CM, or SM and monitored using phase-contrast microscopy; (**B**) Graphs represent the percentages of NSC34 cells showing neurites and the mean lengths of the longest neurites in different culture conditions; (**C**) RT-qPCR analyses of the *BDNF*, *GDNF* and *NGF* genes in T-MSCs, T-MSC-NSs, T-MSC-SCs, and human Schwann cells (HSC). Expression levels were normalized against expression of the housekeeping gene encoding glyceraldehyde 3-phosphate dehydrogenase (GAPDH), and the results are reported as ratios of the marker gene expression versus undifferentiated T-MSCs. Data are presented as the mean ± SE of at least three experiments. Statistical analysis used one-way ANOVA followed by Newman–Keuls multiple comparison tests (* *p* < 0.05; ** *p* < 0.01; *** *p* < 0.001).

SCs secrete several soluble growth factors, which can stimulate neurite outgrowth [22,23]. We used NSC34 mouse motor neuron cells to evaluate whether CM collected from T-MSC-SC cultures could stimulate neurite outgrowth. To eliminate any other effects of Schwann cell induction media, including several molecules such as forskolin, PDGF, bFGF, and heregulin-β1, CM samples were collected after two washes with PBS. As an additional control, NSC34 cells were also cultured in the SC differentiation medium (SM). After being cultured in the CM and SM for four days, some of the NSC34 cells showed neurite outgrowth and their morphological changes were similar to the cells grown in conventional NSC34 differentiation medium (DM), whereas there was no neurite outgrowth of the cells cultured in proliferation medium (PM) (Figure 5A). DM includes effective amounts of all-trans retinoic acid (atRA) and nonessential amino acids (NEAA), which are known to be involved in neuronal outgrowth by regulating the transcriptional level of neurotrophin receptors or other neurite-regulating factors [24,25]. The length of the longest neurite was greater in SM compared with CM. Heregulin in SM might enhance the neurite outgrowth of NSC34 cells [26]. Among the other factors that are present in SM, bFGF is also known to enhance neurite outgrowth by stimulating the MEK-ERK1/2 and PI3K-AKT pathways [27].

Neurite outgrowth was assessed using two independent parameters: the percentage of cells with neurites and the length of the longest neurite (Figure 5B). No NSC34 cells in PM showed extended neurites, but this was significantly increased to 28.24% ± 14.06% (*p* < 0.01) in the CM. The mean maximum length of neurites cultured was 170.33 ± 40.83 µm (*p* < 0.01) in DM, 105.00 ± 20.00 µm (*p* < 0.05) in CM, and 252.70 ± 29.81 µm (*p* < 0.001) in SM.

The expression levels of neurotrophic factors (brain-derived neurotrophic factor (*BDNF*) and glial-cell-derived neurotrophic factor (*GDNF*)) in T-MSC-SCs were increased, whereas the level of nerve growth factor (*NGF)* was decreased compared with their expression levels in the undifferentiated T-MSCs (Figure 5C). However, the expression levels of these neurotrophic factors, including *NGF*, were greatly increased compared with that observed in primary human Schwann cells (HSCs).

### 2.5. T-MSC-SCs Could Myelinate Axons In Vitro

**Figure 6:**
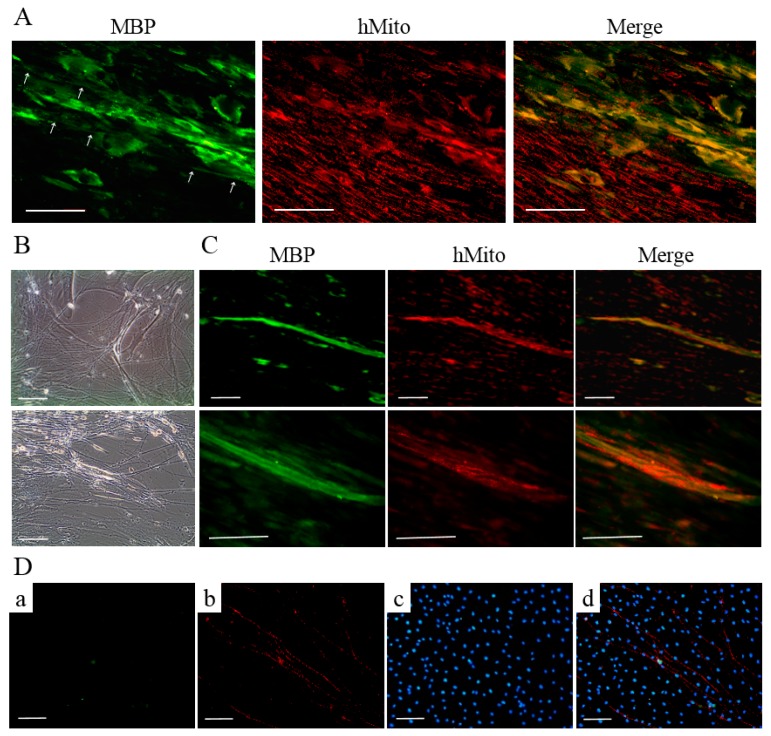
DRG neurons cocultured with T-MSC-SCs: (**A**) immunocytochemical staining of human mitochondria (red) and MBP (green) represent the MBP-positive, but nonmyelinating T-MSC-SCs that are assembled around the small-caliber axons (white arrow); (**B**) phase-contrast images of the association between T-MSC-SCs and neurite bundles extending from mouse DRG explants; and (**C**) immunocytochemical staining for human mitochondria (red) and MBP (green) indicate the formation of myelin sheaths by T-MSC-SCs. Lower rows are enlargements of the myelinated areas of images in the upper row; (**D**) Immunocytochemistry of DRG neurons cultured without T-MSC-SCs did not show MBP-positive axons ((**a**) MBP; (**b**) NF-H; (**c**) DAPI; (**d**) Merge). Scale bar = 100 μm.

**Figure 7:**
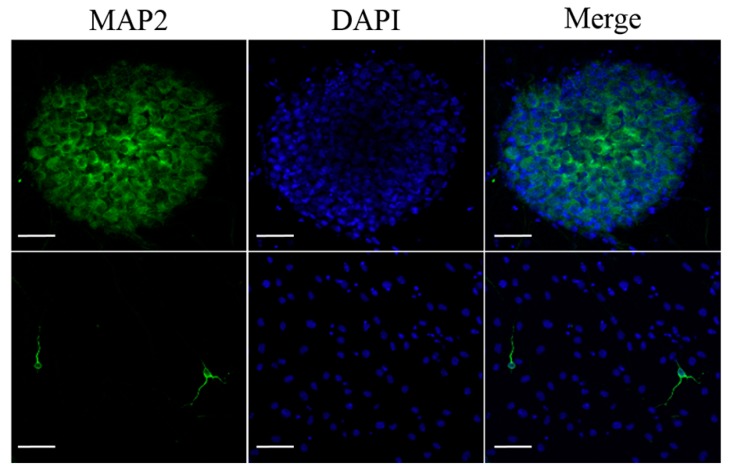
Dendritic marker staining of DRG neurons. Dendritic marker MAP2 immunostaining of sensory neurons in the DRG (**upper** row) and sensory neurons migrating from the DRG (**lower** row). Scale bar = 50 µm.

T-MSC-SCs were cocultured with DRG neurons from mouse embryos after being seeded into a new culture dish. Based on a previous study [28], we applied two sequentially different media formulations to T-MSC-SCs and DRG cocultures. After 4–5 days, extensive neurite projections appeared from the DRG explants and extended well into the regions of T-MSC-SCs; moreover, some of the T-MSC-SCs were associated with bundles of neurites (Figure 6A,B). MBP-positive but nonmyelinating T-MSC-SCs were detected around the small-caliber axons (Figure 6A). However, these T-MSC-SCs were on the outside of the bundles rather than being spread longitudinally along the neurites. After 3–4 weeks, T-MSC-SCs occasionally wrapped around the axons and this formation of myelin sheaths was confirmed by double immunostaining with anti-human mitochondria and anti-MBP antibodies (Figure 6C). The ratio of myelinating events among axons from the DRG coculture with T-MSC-SCs was 14.44% ± 3.84%. MBP-positive axons were not observed when DRG neurons were cultured without T-MSC-SCs (Figure 6D). These results indicate that the DRG neuron preparation did not contain contaminating SCs that might contribute to the myelination process. For dendritic marker staining, MAP2 was positively stained in sensory neurons from the DRG and in migrating sensory neurons from the DRG (Figure 7). However, MAP2-positive cells were not observed in axons or axon bundles.

### 2.6. Functional Recovery of Injured Sciatic Nerve

**Figure 8:**
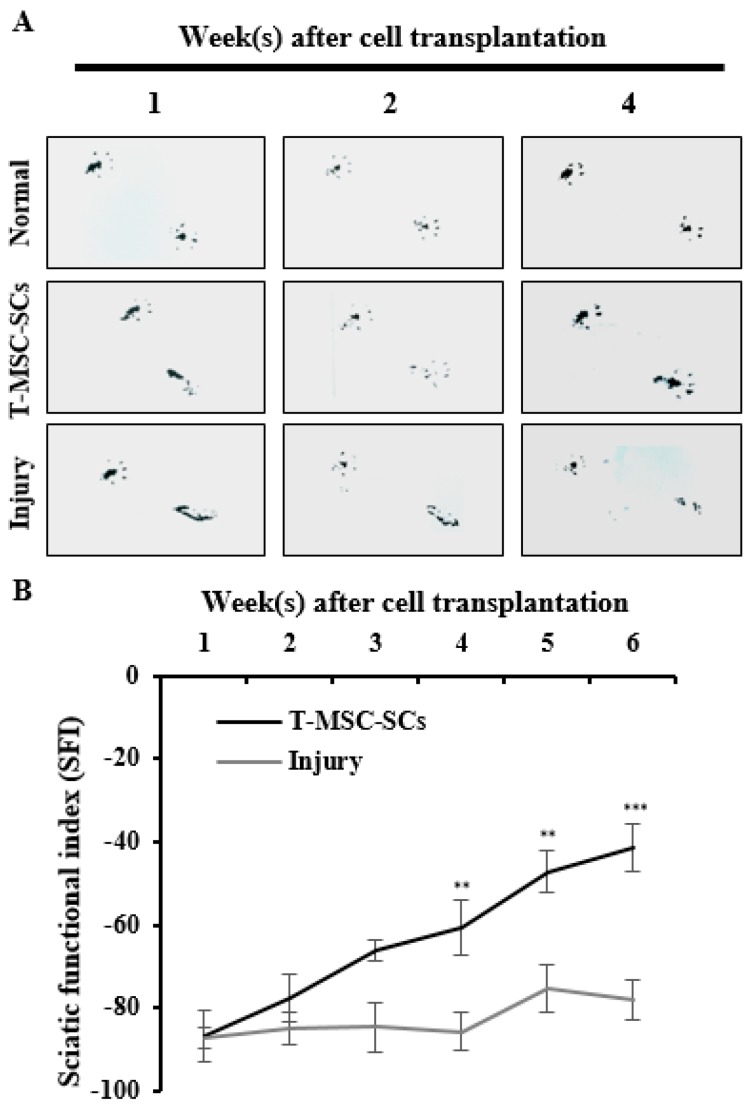

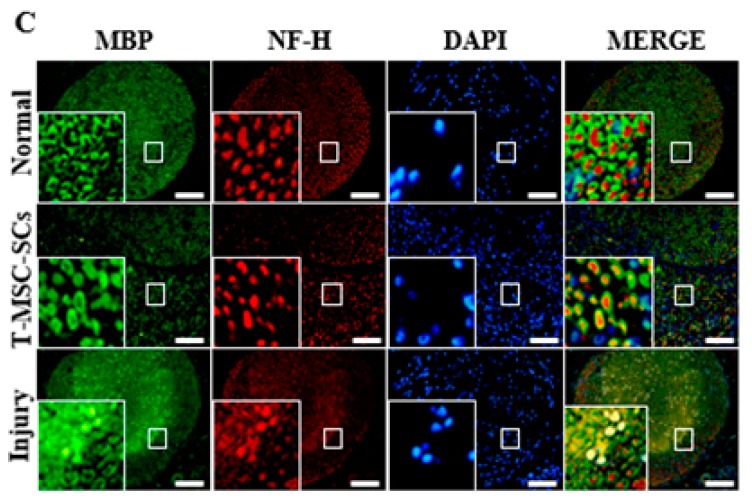
Mouse footprints and immunohistochemistry of the mouse sciatic nerve: (**A**) Mouse footprints at one two and four weeks after cell transplantation. The lower footprints represent the injured side in the T-MSC-SC transplantation group (T-MSC-SCs), injured control group (injury), and non-injured control (normal). Toes were clearly separate from Week 2 in the transplantation group. No significant change was observed in the injury group; (**B**) The SFI from footprinting analysis six weeks after surgery (*n* = 6 for each group). Statistical analyses included two-way ANOVA with Bonferroni post hoc tests (** *p* < 0.01; *** *p* < 0.001); (**C**) Immunohistochemistry of the mouse sciatic nerve six weeks after surgery. Immunofluorescent double staining of sciatic nerve tissue was performed with antibodies to MBP (green, a myelin marker), NF-H (red, an axonal marker) and DAPI (blue). The transplantation group showed regenerating axons surrounded by SCs. Original magnification 400×. Scale bar = 200 µm.

To evaluate the efficacy of T-MSC-SCs in ameliorating a mouse model of peripheral nerve injury, T-MSC-SC grafts were transplanted to the scarred region of the mouse sciatic nerve following partial sciatic nerve axotomy. Analyses of functional recovery were performed weekly by studying footprint patterns (Figure 8A). Up to one week after transplantation, toe arrangements were unaltered in the cell transplantation and injury groups compared with the control group. By Week 2, the transplantation group revealed a marked improvement in toe separation, which was sustained for the duration of the experiment. Although the transplantation group showed radical improvements in gait, the injured and untreated mice did not show any changes in toe separation. These measurements were calculated according to the SFI formula (Figure 8B) [29].

**Figure 9:**
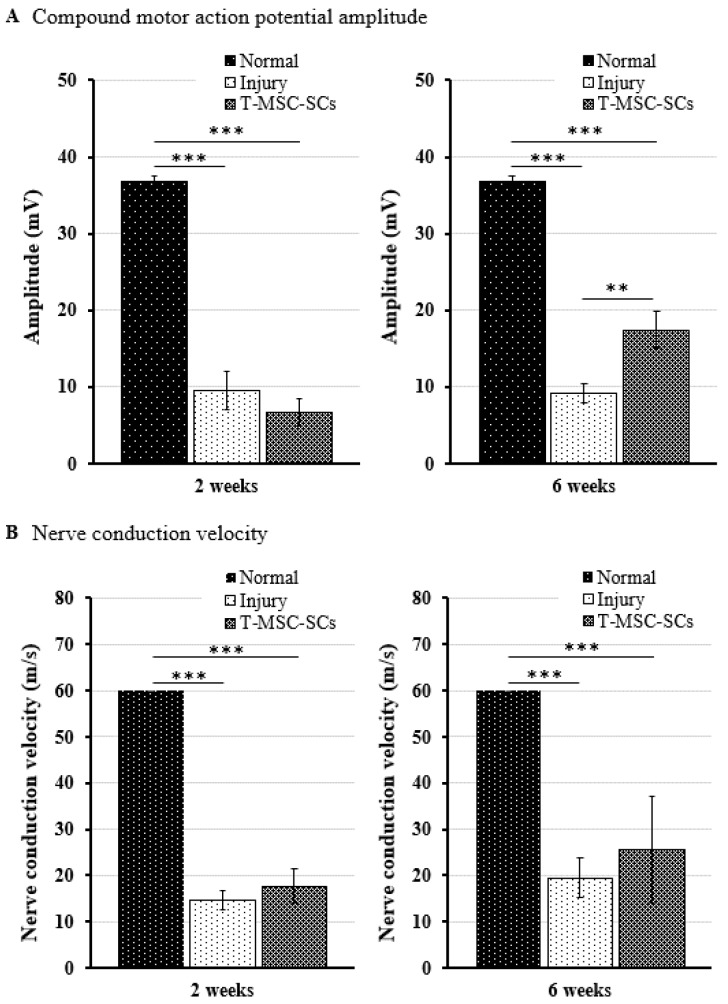
For electrophysiological analyses, the compound motor action potential (CMAP) amplitudes and motor nerve conduction velocity (NCV) were determined using Nicolet VikingQuest. Comparison of the CMAP amplitudes (**A**) and NCV (**B**) of the operated side in the injury and transplantation groups at two and six weeks after surgery. The CMAP amplitude and NCV of the non-injured control (normal) groups were 36.74 ± 0.82 mV and 60 ± 0 m/s, respectively. Statistical analyses were performed using two-way ANOVA with Bonferroni post hoc tests (** *p* < 0.01; *** *p* < 0.001).

After gait analysis, immunohistochemistry was performed to evaluate the regeneration efficacy of myelinated nerves (Figure 8C). Immunostaining showed that numerous regenerated axons were surrounded by myelin sheaths, and some of those axons formed clusters in the transplantation group. In the injury group, some of the myelin sheaths appeared distorted, and many axons were disrupted. To verify these results, we used electrophysiological measures of compound muscle action potential (CMAP) amplitude and motor nerve conduction velocity (NCV) (Figure 9). Figure 9 shows a statistically significant (*p* < 0.01) increase in the CMAP amplitudes of the T-MSC-SC transplantation group (17.41 ± 2.37 mV) compared with the injury group (9.10 ± 1.23 mV) at six weeks after operation. However, there was no significant difference in MNC velocity between the injury and transplantation groups of mice. Because CMAP amplitude is dependent on the number of axons, these data suggest that T-MSC-SCs had the ability to support regeneration of the sciatic nerve [30].

## 3. Discussion

Here, we confirmed that T-MSCs isolated from human palatine tonsils have the ability to differentiate along a glial cell lineage and express cell markers that are typical for glial cells including SCs. Using RT–qPCR and Western blot analyses, we observed the expression of SC-specific markers. The expression of immature SC markers, GFAP and NGFR, were increased in T-MSC-SC but GFAP expression was also shown in undifferentiated T-MSCs to a lesser extent. This observation was consistent with a report that BMSCs could acquire GFAP expression after 4–5 passages without any particular neural induction [31]. The highest expression of an immediate early gene, *KROX20*, was observed at neurosphere stage. However, the expression of another immediate early gene, *KROX24*, was highest in T-MSC-SCs. In accordance with that reported by other studies, *KROX20 * and *KROX24* were expressed in a successive and mutually exclusive manner [32,33,34]. Krox20 promotes the differentiation of SCs to a myelinating phenotype while Krox24 is a non-myelinating SC marker [33]. Krox20 and Krox24 are playing antagonistic roles during the development of the SC lineage [34].

During SC development, mature SCs finally differentiate into two different functional categories: myelinating and nonmyelinating types. Myelinating SCs selectively wrap large-diameter axons, while nonmyelinating SCs occasionally attach to small neuronal bundles [4]. To investigate whether T-MSC-SCs could acquire these dual capacities of mature SCs, we performed coculture with primary DRG neurons isolated from 12.5 to 13-day-old mouse embryos. After 4–5 days, we observed that nerve fibers from the DRG explants had a tendency to grow toward nearby T-MSC-SCs and attached to some of them by their tips. Some of these formed bundles, but their association patterns were not considered as indicating elongated myelination. Myelin sheaths newly formed by T-MSC-SCs were observed after 3–4 weeks and verified through double staining with anti-human mitochondria and anti-MBP antibodies. MBP is a specific marker of the mature stage of SC development and is a main component of myelin [35]. Liu et al. reported that the MBP expression rate was high in primary SC pure cultures at the early stage, and reached 100% at P3 SCs [35]. However, the expression level of MBP in differentiated T-MSC-SCs per se was decreased in our study. As shown in Figure 6A, MBP expression in T-MSC-SCs was only detected when the T-MSC-SCs made contact with the DRG neurons. The expression of MBP might depend on SC–axon communication, and myelination by SCs is entirely dependent on the establishment of contact with axons [36]. Although we could not measure the myelination efficacy or functional relevance of these T-MSC-SCs, this observation indicates that the biology of these T-MSC-SCs was functionally analogous to endogenous SCs, so that they might be suitable for cell transplantation in cases of peripheral nerve injury.

Mature SCs are well known to produce soluble neurotrophic factors that support the growth of axons, including NGF, BDNF, ciliary neurotrophic factor, neurotrophin-3, and FGF [1,12]. We collected CM from T-MSC-SC cultures and investigated its possible beneficial effects on neurite outgrowths of NSC34 mouse motor neurons. The percentages of cells with neurites and the lengths of the longest neurites showed similar effects to conventional NSC34 neurite DM containing NEAA and atRA. We observed that the expression levels of *BDNF* and *GDNF* were much higher than those detected in the undifferentiated T-MSCs and in primary HSCs. Thus, neurite outgrowth might be controlled by soluble neurotrophic factors secreted by T-MSC-SCs. Initially, we tried to use NSC34 cells not only for neurite outgrowth, but also in coculture experiments. However, in contrast with the neurite outgrowth experiments, the NSC34 cells could not be used to perform coculture experiments because of the limitations relating to the cell size and length of axonal outgrowth of NSC34. Therefore, we used the DRG explants for coculture with T-MSC-SCs insisted of NSC34 cells. We observed that T-MSC-SCs were able to myelinate axons during coculture with DRG explants.

Here, we demonstrated that T-MSC-SCs significantly improved the gait in injured mice compared with untreated groups, and this suggests that T-MSC-SCs supported robust axon outgrowth and structural formation of myelin sheaths in this rodent model of acute peripheral nerve injury. Consistent with the footprint results, the pattern of immunostaining of MBP and NF-H proteins in the regenerating sciatic nerves and increased CMAP amplitudes indicated that T-MSC-SCs facilitated axonal regrowth and remyelination. As it is known that regenerated nerves are often smaller in diameter with thinner myelin sheaths than normal nerves, the regenerated axons in mice with the T-MSC-SC transplants also showed relatively smaller axons and thinner myelin than normal [37]. However, the myelin sheath structure revealed a rigid structure compared with the untreated injured group. As T-MSC-SCs were able to myelinate axons during coculture with DRG explants, we hypothesized that T-MSC-SCs improve the gait after nerve injury repair by myelinating the regenerated axons directly. However, human-specific markers such as anti-human mitochondria or anti-human nuclei antibodies were barely detected after T-MSC-SC transplantation into the mice with peripheral nerve injury. One explanation as to how these transplants could induce such a remarkable recovery is that they might recruit endogenous host SCs to undertake myelination, as shown in a model of spinal cord injury [38]. This positive effect may be caused by the neurotrophic actions of the endogenous host SCs. Another strong possibility is that the secretion of neurotrophic factors from the T-MSC-SCs may affect axonal regeneration in the injured sciatic nerve. In accordance with neurite outgrowth experiments, high expression level of BDNF and GDNF observed in the T-MSC-SCs might play important roles in this regeneration process. T-MSC-SCs may provide a more suitable environment for axonal regeneration by providing neurotrophic factors, thereby helping regenerating axons to avoid cues that are nonpermissive regrowth [39]. Although damaged peripheral nerve axons have the capacity to regrow, functional recovery is often incomplete. This is because axonal growth cones can be misdirected by encountering physical barriers such as glial cells, inflammatory cells, and myelin debris during the regeneration process. Besides their neurotrophic and myelination functions, SCs play critical roles in degrading tissue debris at the injury site and provide a trophic environment for nerve regeneration [2]. Thus, given these diverse roles of SCs, T-MSC-SCs might induce functional improvements in our model of MSC-induced peripheral nerve repair following injury.

Until now, T-MSCs have been mainly studied to understand their basic characteristics as MSCs. According to the reports by our group and others, T-MSCs exhibit typical expression patterns of MSC surface markers (negative for CD14, CD34, and CD45; positive for CD73, CD90, and CD105) and have multilineage differentiation potential [14,15,16,17,40]. Previous studies also emphasized the relatively short doubling time of T-MSCs (about 38 h) compared with other types of MSCs (14–17). This study is the first demonstration that human T-MSCs can be differentiated into SCs under appropriate conditions. When combining our data with previous studies, it appears that the differentiation potential of T-MSCs can overcome differentiation limits so that they can enter mesodermal, endodermal, and ectodermal lineages.

In conclusion, we have shown here that T-MSCs have the capacity to differentiate into SCs. Their expression of SC-specific markers, support of neurite outgrowth, and formation of myelin sheaths indicate that T-MSC-SCs have capacities that are similar to those of endogenous SCs. T-MSC-SC transplantation produced functional improvements in a mouse model of sciatic nerve injury. Therefore, T-MSCs may serve as a valuable cell source for SC transplantation, and the transplantation of human T-MSC-SCs may be suitable for assisting in peripheral nerve regeneration. However, the injury model used in this study was a mild injury model. Further studies of the application of T-MSC-SCs in other models with moderate or severe injury, such as the standard 15-mm nerve defect model, will provide additional information regarding the viability of the use of T-MSC-SCs in peripheral nerve regeneration.

## 4. Materials and Methods

### 4.1. Tonsil-Derived Mesenchymal Stem Cell (T-MSC) and Human Schwann Cell (HSC) Culture

T-MSCs were isolated and cultured as we described previously [15,17], with minor modifications. In brief, in this study we used tonsils obtained from one patient aged 6 years undergoing tonsillectomy. Informed written consent was obtained from legal guardian of the patient who participated in this study, and the study protocol was approved by the Institutional Review Board (ECT-11-58-37) of Ewha Womans University, Mokdong Hospital (Seoul, Korea). Isolated tonsillar tissues were washed three times with phosphate-buffered saline (PBS), chopped, and incubated with collagenase type I (210 U/mL; Gibco BRL, Carlsbad, CA, USA) and DNase I (10 µg/mL; Sigma-Aldrich, St. Louis, MO, USA) in 10 mL Dulbecco’s Modified Eagle’s Medium (DMEM, Hyclone, Logan, UT, USA) for 30 min at 37 °C with stirring. Digested tissues were filtered through a cell strainer (pore size 70 µm; SPL, Pocheon, Korea) and cells were harvested and washed twice by centrifugation at 300× *g* for 3 min at room temperature. Among these cells, we obtained mononuclear cells (MNCs) using a Ficoll–Paque™ PREMIUM (GE Healthcare, Pittsburgh, PA, USA) density gradient centrifugation at 300× *g* for 30 min at room temperature. MNCs were plated at a density of of 1 × 10^8^ cells per T-150 flask in DMEM supplemented with 10% FBS, and 1% penicillin/streptomycin (Hyclone) under humidified 5% CO_2_ in air at 37 °C. After 48 h, nonadherent cells were removed, and the remaining adherent cells (hereafter called T-MSCs) were cultured in DMEM growth medium and subcultured twice per week. T-MSCs were confirmed based on specific surface antigen expression: no expression of the hematopoietic stem cell biomarkers CD14, CD34, and CD45, and positive expressions of the mesenchymal stem cell biomarkers CD73, CD90, and CD105. All T-MSCs used in this study were between passages 6 and 8.

HSCs were purchased from ScienCell (cat. no. 1700, Carlsbad, CA, USA) and cultured in media (cat. no. 1701) according to the manufacturer’s instruction in a humidified incubator with 5% CO_2_ at 37 °C. Media were changed every 3 days.

### 4.2. Adipogenic, Chondrogenic and Osteogenic Differentiation

The mesodermal differentiation of T-MSCs was examined, as described [17]. For an adipogenic differentiation, T-MSCs were cultured in commercially available adipogenic media (Invitrogen Life Technologies, Carlsbad, CA, USA) for 3 weeks. After washing in PBS, they were fixed in 4% paraformaldehyde for 15 min, then washed again with PBS and stained with 2% Oil Red O (Sigma-Aldrich) for 1 h at room temperature. T-MSCs were rinsed with PBS again. Intracellular lipid droplets were observed by light microscopy. For chondrogenic differentiation, T-MSCs were cultured for 3 weeks in chondrogenesis-inducing medium (Invitrogen Life Technologies). The cells were washed with PBS and fixed in 4% paraformaldehyde for 15 min. After another wash with PBS, they were stained with 1% Alcian blue (Sigma-Aldrich) for 1 h at room temperature. To remove the excess dye, T-MSCs were washed again with PBS. After the cells had been rinsed with 0.1 N HCl, they were observed using phase-contrast microscopy. For osteogenic differentiation, T-MSCs were cultured in osteogenic media (Invitrogen) for 3 weeks. Thereafter, they were washed with PBS and fixed in 4% paraformaldehyde for 15 min. After staining with 2% Alizarin Red S (Sigma-Aldrich) for 1 h, the cells were washed twice again with PBS. Extracellular matrix calcification was visualized by microscopy.

### 4.3. Animals and Transplantation with T-MSC-SCs

The use and care of experimental animals were approved by the Institutional Animal Care and Use Committee at Ewha Womans University School of Medicine (ESM#15-0294), and all experiments were performed in accordance with the approved guidelines and regulations. For the injury control group, adult male C57BL/6 mice weighing 20–30 g were anesthetized (intraperitoneal injections of 50 mg/kg Zoletil, Virbac, Carros, France) and the right sciatic nerve was partially transected at the sciatic notch to form an interneural scar. For T-MSC-SC transplantation, differentiated cells were suspended in 6% poly(ethylene glycol)-b-poly(l-alanine) (PEG-L-PA) gel (kindly provided by Byeongmoon Jeong, Department of Chemistry and Nano Science, Ewha Womans University, Seoul, Korea), dissolved in DMEM/F12 supplemented with 50 mg/mL l-ascorbic acid at a concentration of 4 × 10^6^ cells/mL, and transferred into a 7-mm PVC tube following nerve surgery. Six mice were used in each of the normal control and T-MSC-SC transplantation groups, and five were used as injured untreated controls.

### 4.4. Differentiation to a SC Phenotype

To differentiate T-MSCs into SCs, we used the technique of Razavi et al. [41] in which human T-MSCs were induced to form neurospheres. We harvested human T-MSCs (80%–90% confluence) and then plated them in plastic dishes at (1.5–2) × 10^5^ cells/cm^2^ in DMEM/F-12 (Welgene Inc., Daegu, Korea) supplemented with 20 ng/mL basic fibroblast growth factor (bFGF, PeproTech, London, UK), 20 ng/mL epidermal growth factor (EGF, PeproTech), and 2% B27 supplement (1:50, Gibco, Life Technologies, Burlington, ON, Canada) at 37 °C under 5% CO_2_ in humidified air. We replenished the cultures with fresh medium every 3–4 days. After 7 days, neurospheres were triturated using a 25-gauge needle and replated in laminin-coated cell culture plates containing DMEM/F12 supplemented with 10% FBS, 14 µM forskolin (Sigma-Aldrich), 5 ng/mL platelet-derived growth factor-AA (PDGF, PeproTech), 10 ng/mL bFGF (PeproTech) and 200 ng/mL recombinant human heregulin-β1 (PeproTech) for terminal differentiation. The cells were incubated for 9 days under these conditions, and then harvested for further investigations.

### 4.5. RT-qPCR

This was performed using SYBR^®^ Premix Ex Taq™ kits (TaKaRa Bio Inc., Shiga, Japan) on an ABI 7500 Fast Real-Time PCR system (Applied Biosystems/Thermo Fisher Scientific, Waltham, MA, USA) to confirm the relative expression levels of genes in T-MSC and T-MSC-SC cell lines. The following human primers were used: forward *GAPDH* primer: 5′-CCCACTCCTCCACCTTTGAC-3′, reverse *GAPDH* primer: 5′-CTGTTGCTGTAGCCAAATTCG-3′; forward *CAD19* primer: 5′-TTACTGCTGCGTTTTATGTTGGG-3′, reverse *CAD19* primer: 5′-CCAGCCACGCTTCACTCTC-3′; forward *GFAP* primer: 5′-CCGACAGCAGGTCCATGTG-3′, reverse *GFAP* primer: 5′-GTTGCTGGACGCCATTGC-3′; forward *KROX20* primer: 5′-AACGGAGTGGCCGGAGAT-3′, reverse *KROX20* primer: 5′-ATGGGAGATCCAACGACCTCTT-3′; forward *KROX24* primer: 5′-CAGCAGTCCCATTTACTCAG-3′, reverse *KROX24* primer: 5′-GACTGGTAGCTGGTATTG-3′; forward *MBP* primer: 5′-ATCCAAGTACCTGGCCACAG-3′, reverse *MBP* primer: 5′-CAAGGATGCCCGTGTCTC-3′; forward *NGFR* primer: 5′-CCTACGGCTACTACCAGGAT-3′, reverse *NGFR* primer: 5′-TGGCCTCGTCGGAATACG-3′; forward *S100B* primer: 5′-GGAGACGGCGAATGTGACTT-3′, reverse *S100B* primer: 5′-GAACTCGTGGCAGGCAGTAGTAA-3′; forward *BDNF* primer: 5′-GATGCCAGTTGCTTTGTCTTC-3′, reverse *BDNF* primer: 5′-TAAAATCTCGTCTCCCCAACA-3′; forward *GDNF* primer: 5′-TTCAAGCCACCATTAAAAGAC-3′, reverse *GDNF* primer: 5′-ATAGCCCAGACCCAAGTCAGT-3′; forward *NGF* primer: 5′-GTCAGCGTGTGGGTTGGGGATA-3′, reverse *NGF* primer: 5′-GACAAAGGTGTGAGTCGTGGT-3′. Relative gene expression was analyzed using the comparative *C*_t_ method ($2^{-\Delta \Delta C_{t}}$) [42]. All measurements were carried out in triplicate.

### 4.6. Western Blot Analysis

T-MSCs and T-MSC-SCs were washed with ice-cold PBS and lysed in PRO-PREP buffer containing a phosphatase inhibitor cocktail solution (iNtRON Biotechnology, Seongnam-si, Korea) for 30 min on ice. After centrifugation at 13,000× *g* for 20 min at 4 °C, equal quantities of protein from supernatants were separated by sodium dodecyl sulfate–polyacrylamide gel electrophoresis (SDS–PAGE) and were electrophoretically transferred onto polyvinylidene membranes (Millipore, Billerica, MA, USA). The blots were then probed overnight at 4 °C with antibody against the glial fibrillary acidic protein (GFAP) (1:400, monoclonal antibody, Sigma-Aldrich, cat. no. G3893) or the nerve growth factor receptor (NGFR/p75) (1:500, polyclonal antibody, Santa Cruz Biotechnology, cat. no. sc8317, Dallas, TX, USA), followed by the corresponding secondary antibody. The blots were washed and developed using enhanced chemiluminescence reagents (WestSave GOLD™ Western Blot Detection kits) (AbFrontier, Seoul, Korea), according to the manufacturer’s instructions. Band intensities were assessed by densitometric scanning (LAS-3000, Fujifilm, Tokyo, Japan).

### 4.7. Immunocytochemistry and Immunohistochemistry

T-MSCs and T-MSC-SCs were trypsinized and added to laminin-coated cell culture slides. The cells were fixed in 4% (*w*/*v*) paraformaldehyde (15 min, room temperature) and washed three times in ice-cold PBS. After blocking with 1% bovine serum albumin (BSA, Bovogen Biologicals, Melbourne, VIC, Australia), the cells were incubated overnight at 4 °C with an anti-GFAP monoclonal antibody (1:200, Sigma-Aldrich, cat. no. G3893) or an anti-NGFR polyclonal antibody (1:200, Santa Cruz Biotechnology, cat. no. sc8317). After rinsing in PBS, secondary goat anti-mouse antibodies and secondary goat anti-rabbit antibodies, both conjugated with Alexa Fluor 488, were applied for 1 h at room temperature in the dark. The cells were mounted with Vectashield 1 (Vector Laboratories, Burlingame, CA, USA) mounting medium containing 4′,6-diamidino-2-phenylindole (DAPI). The cells were observed using an Olympus BX51 phase-contrast microscope (Tokyo, Japan).

For immunohistochemistry, mouse sciatic nerves were fixed in 10% formaldehyde. Following approximately 24 h of fixation at 4 °C, the nerves were washed in PBS at room temperature. The nerves were dehydrated in a graded ethanol series, cleared in xylene, and embedded in paraffin wax. The blocks were sectioned into 5-µm thick serial sections. Dewaxed sections were treated with 3% hydrogen peroxide for 20 min to block endogenous peroxidase and incubated in a microwave with 0.01 M citric acid buffer (pH 6) for three cycles of 5 min each at 850 W for antigen retrieval. Subsequently, the sections were blocked with 20% horse serum (Hyclone) in PBS for 1 h at room temperature, then incubated with an anti-myelin basic protein (MBP) polyclonal antibody (1:100, Millipore, cat. no. AB980) and an anti-neurofilament heavy polypeptide (NF-H) monoclonal antibody (1:100, Santa Cruz Biotechnology, cat. no. sc58553) at 4 °C overnight. After rinsing in PBS, secondary goat anti-mouse antibodies conjugated with Alexa Fluor 568 and secondary goat anti-rabbit antibodies conjugated with Alexa Fluor 488 were applied for 1 h at room temperature in the dark. The cells were mounted using Vectashield 1 (Vector Laboratories) mounting medium containing DAPI.

### 4.8. CM Preparation

CM was collected from T-MSC-SC cultures were grown to 80% confluency. After aspirating the culture medium and washing the cells twice with PBS, cells were further incubated with DMEM supplemented with 2% FBS at 37 °C in 5% CO_2_ in humidified air. After 2 days, we harvested and centrifuged the medium at 1000× *g* for 5 min, and collected the supernatant as CM.

### 4.9. Assessment of the Differentiation of NSC34 Cells

Mouse motor neuron-like cell line NSC34 cells seeded 1 mL aliquots of suspensions containing 2 × 10^4^ cells/mL in each well of 6-well plates coated with poly-l-lysine. Twenty-four hours later, we washed the cells twice with PBS and cultured them for 4 days in the following media: (1) DMEM with 10% FBS (proliferation medium group); (2) DMEM: F12 (1:1) with 1% FBS, 1% modified Eagle’s medium containing nonessential amino acids (NEAA), 1 µM all-trans retinoic acid (NSC34 neurite differentiation medium group); and (3) CM (T-MSC-SC CM group). A cell with a neurite length >50 µm was regarded as differentiated.

### 4.10. T-MSC-SCs and Mouse DRG Cell Coculture

For evaluating the degree of myelination in cocultures, confluent cultures of T-MSC-SCs were trypsinized and added to laminin-coated 2-well cell culture slides (SPL Lifesciences Inc., Seoul, Korea), 1 day before adding DRGs. To purify DRG neurons, pregnant ICR mice were purchased from Korean BioLink Co. (Chungbuk, Korea). Gestational Day 12.5–13 embryos were removed and DRGs were dissected out. These were washed gently twice with 1 mL of DMEM with 10% FBS, then plated directly onto the slides seeded with T-MSC-SCs. They were then incubated for 4 days in coculture medium: Eagle’s basal medium, ITS supplement, 0.2% BSA, 4 mg/mL d-glucose (all from Sigma-Aldrich), Glutamax (Gibco), 50 ng/mL nerve growth factor (NGF, PeproTech), and antibiotics, and then switched to the same medium supplemented with 15% FBS and 50 mg/mL l-ascorbic acid (Sigma-Aldrich) for an additional 3–4 weeks to induce myelination. This was based on the technique of Krause et al. with slight modification [28].

For immunostaining, cells on the chamber slides were fixed in 4% (*w*/*v*) paraformaldehyde (15 min, room temperature) and washed three times in ice-cold PBS. After blocking with 1% BSA (Bovogen), cocultured cells were incubated overnight at 4 °C with an anti-MBP polyclonal antibody (1:200, Millipore, cat. no. AB980) and an anti-human mitochondria antibody (1:400, Millipore, cat. no. MAB1273); DRG control cells were incubated with an anti-MAP2 polyclonal antibody (1:400, Millipore, cat. no. AB5622). After rinsing in PBS, secondary goat anti-rabbit antibodies conjugated with Alexa Fluor 488 and secondary goat anti-mouse antibodies conjugated with Alexa Fluor 568, were applied for 1 h at room temperature in the dark. The cells were mounted with Vectashield1 (Vector Laboratories) mounting medium containing DAPI. The cells were observed using an Olympus BX51 fluorescence microscope (Tokyo, Japan).

### 4.11. Footprint Analysis of Gait and Evaluation of Sciatic Functional Index (SFI)

The normal and injured hind paws were painted with black dye and the mice were encouraged to walk in a straight line along an 80 cm long runway over paper. The footprint patterns were then observed. A series of at least five sequential steps recorded in the same session was used to determine the walking pattern of each mouse. After inducing the injury, animals were tested once a week for 6 weeks. For quantitative analysis, footprints were evaluated with the Footprint analysis of gait and evaluation of sciatic functional Index (SFI), as described [29]. The parameters of toe spread and paw-print length from the intact and injured or transplanted sides were assessed to calculate the SFI.

### 4.12. Electrophysiological Studies

Four of each of the injured and T-MSC-SCs transplanted mice were used for electrophysiological studies at 6 weeks after the operation. The mice were lightly anesthetized with isoflurane and the fur from the distal back and hind limbs was removed completely. The CMAP amplitudes and motor NCV were determined using Nicolet VikingQuest (Natus Medical, San Carlos, CA, USA) [30]. The sciatic-tibial motor NCV was determined by recording at the dorsum of the foot with stimulation applied first at the ankle, then at the sciatic notch. Latencies were measured from the initial onset of the CMAP. Final NCV was determined by dividing the difference of the ankle from the notch distance by the difference between the ankle and notch latencies.

### 4.13. Statistical Analysis

The values are presented as the mean ± standard deviation or ± standard error (SE). Data were analyzed by one-way or two-way analysis of variance (ANOVA) with further post hoc tests using the statistical software of GraphPad Prism version 4 (GraphPad Software, Inc., San Diego, CA, USA). Differences in results between NSC34 neurite culture conditions were analyzed by one-way ANOVA with Newman–Keuls multiple comparison tests. A repeated-measures two-way ANOVA, with Bonferroni post hoc tests, was also used to determine any statistically significant differences among non-injured, injured, and transplanted mouse groups. Student’s *t*-test was used to analyze and compare two groups. A *p* value of <0.05 was considered as statistically significant for each experiment.

## References

[1] Monk K.R., Feltri M.L., Taveggia C. (2015). New insights on schwann cell development. Glia.

[2] Gaudet A.D., Popovich P.G., Ramer M.S. (2011). Wallerian degeneration: Gaining perspective on inflammatory events after peripheral nerve injury. J. Neuroinflamm..

[3] Lee Y.C., Chang M.H., Lin K.P. (2008). Charcot-Marie-Tooth disease. Acta Neurol. Taiwan.

[4] Pereira J.A., Lebrun-Julien F., Suter U. (2012). Molecular mechanisms regulating myelination in the peripheral nervous system. Trends Neurosci..

[5] Viader A., Sasaki Y., Kim S., Strickland A., Workman C.S., Yang K., Gross R.W., Milbrandt J. (2013). Aberrant schwann cell lipid metabolism linked to mitochondrial deficits leads to axon degeneration and neuropathy. Neuron.

[6] Ame-Thomas P., Maby-El Hajjami H., Monvoisin C., Jean R., Monnier D., Caulet-Maugendre S., Guillaudeux T., Lamy T., Fest T., Tarte K. (2007). Human mesenchymal stem cells isolated from bone marrow and lymphoid organs support tumor B-cell growth: Role of stromal cells in follicular lymphoma pathogenesis. Blood.

[7] May F., Weidner N., Matiasek K., Caspers C., Mrva T., Vroemen M., Henke J., Lehmer A., Schwaibold H., Erhardt W. (2004). Schwann cell seeded guidance tubes restore erectile function after ablation of cavernous nerves in rats. J. Urol..

[8] Williams A.R., Hare J.M. (2011). Mesenchymal stem cells: Biology, pathophysiology, translational findings, and therapeutic implications for cardiac disease. Circ. Res..

[9] Grochmal J., Dhaliwal S., Stys P.K., van Minnen J., Midha R. (2014). Skin-derived precursor schwann cell myelination capacity in focal tibial demyelination. Muscle Nerve.

[10] Reid A.J., Sun M., Wiberg M., Downes S., Terenghi G., Kingham P.J. (2011). Nerve repair with adipose-derived stem cells protects dorsal root ganglia neurons from apoptosis. Neuroscience.

[11] Ladak A., Olson J., Tredget E.E., Gordon T. (2011). Differentiation of mesenchymal stem cells to support peripheral nerve regeneration in a rat model. Exp. Neurol..

[12] Peng J., Wang Y., Zhang L., Zhao B., Zhao Z., Chen J., Guo Q., Liu S., Sui X., Xu W. (2011). Human umbilical cord wharton’s jelly-derived mesenchymal stem cells differentiate into a schwann-cell phenotype and promote neurite outgrowth in vitro. Brain Res. Bull..

[13] Bajada S., Mazakova I., Richardson J.B., Ashammakhi N. (2008). Updates on stem cells and their applications in regenerative medicine. J. Tissue Eng. Regen. Med..

[14] Janjanin S., Djouad F., Shanti R.M., Baksh D., Gollapudi K., Prgomet D., Rackwitz L., Joshi A.S., Tuan R.S. (2008). Human palatine tonsil: A new potential tissue source of multipotent mesenchymal progenitor cells. Arthritis Res. Ther..

[15] Ryu K.H., Cho K.A., Park H.S., Kim J.Y., Woo S.Y., Jo I., Choi Y.H., Park Y.M., Jung S.C., Chung S.M. (2012). Tonsil-derived mesenchymal stromal cells: Evaluation of biologic, immunologic and genetic factors for successful banking. Cytotherapy.

[16] Djouad F., Jackson W.M., Bobick B.E., Janjanin S., Song Y., Huang G.T., Tuan R.S. (2010). Activin a expression regulates multipotency of mesenchymal progenitor cells. Stem Cell Res. Ther..

[17] Yu Y., Park Y.S., Kim H.S., Kim H.Y., Jin Y.M., Jung S.C., Ryu K.H., Jo I. (2014). Characterization of long-term in vitro culture-related alterations of human tonsil-derived mesenchymal stem cells: Role for CCN1 in replicative senescence-associated increase in osteogenic differentiation. J. Anat..

[18] Jessen K.R., Mirsky R. (2005). The origin and development of glial cells in peripheral nerves. Nat. Rev. Neurosci..

[19] Takahashi M., Osumi N. (2005). Identification of a novel type ii classical cadherin: Rat cadherin19 is expressed in the cranial ganglia and schwann cell precursors during development. Dev. Dyn..

[20] Lin J., Luo J., Redies C. (2010). Cadherin-19 expression is restricted to myelin-forming cells in the chicken embryo. Neuroscience.

[21] Jessen K.R. (2004). Glial cells. Int. J. Biochem. Cell Biol..

[22] Kingham P.J., Kalbermatten D.F., Mahay D., Armstrong S.J., Wiberg M., Terenghi G. (2007). Adipose-derived stem cells differentiate into a schwann cell phenotype and promote neurite outgrowth in vitro. Exp. Neurol..

[23] Xu Y., Liu Z., Liu L., Zhao C., Xiong F., Zhou C., Li Y., Shan Y., Peng F., Zhang C. (2008). Neurospheres from rat adipose-derived stem cells could be induced into functional schwann cell-like cells in vitro. BMC Neurosci..

[24] Clagett-Dame M., McNeill E.M., Muley P.D. (2006). Role of all-trans retinoic acid in neurite outgrowth and axonal elongation. J. Neurobiol..

[25] Yasuda E., Ma N., Semba R. (2001). Immunohistochemical demonstration of l-serine distribution in the rat brain. Neuroreport.

[26] Audisio C., Mantovani C., Raimondo S., Geuna S., Perroteau I., Terenghi G. (2012). Neuregulin1 administration increases axonal elongation in dissociated primary sensory neuron cultures. Exp. Cell Res..

[27] Lin W.F., Chen C.J., Chang Y.J., Chen S.L., Chiu I.M., Chen L. (2009). SH2B1β enhances fibroblast growth factor 1 (FGF1)-induced neurite outgrowth through MEK-ERK1/2-STAT3-EGR1 pathway. Cell Signal..

[28] Krause M.P., Dworski S., Feinberg K., Jones K., Johnston A.P., Paul S., Paris M., Peles E., Bagli D., Forrest C.R. (2014). Direct genesis of functional rodent and human schwann cells from skin mesenchymal precursors. Stem Cell Rep..

[29] Inserra M.M., Bloch D.A., Terris D.J. (1998). Functional indices for sciatic, peroneal, and posterior tibial nerve lesions in the mouse. Microsurgery.

[30] Xia R.H., Yosef N., Ubogu E.E. (2010). Dorsal caudal tail and sciatic motor nerve conduction studies in adult mice: Technical aspects and normative data. Muscle Nerve.

[31] Tondreau T., Lagneaux L., Dejeneffe M., Massy M., Mortier C., Delforge A., Bron D. (2004). Bone marrow-derived mesenchymal stem cells already express specific neural proteins before any differentiation. Differentiation.

[32] Jessen K.R., Mirsky R. (2008). Negative regulation of myelination: Relevance for development, injury, and demyelinating disease. Glia.

[33] Balakrishnan A., Stykel M.G., Touahri Y., Stratton J.A., Biernaskie J., Schuurmans C. (2016). Temporal analysis of gene expression in the murine schwann cell lineage and the acutely injured postnatal nerve. PLoS ONE.

[34] Topilko P., Levi G., Merlo G., Mantero S., Desmarquet C., Mancardi G., Charnay P. (1997). Differential regulation of the zinc finger genes *Krox*-20 and *Krox*-24 (*Egr*-1) suggests antagonistic roles in schwann cells. J. Neurosci. Res..

[35] Liu Z., Jin Y.Q., Chen L., Wang Y., Yang X., Cheng J., Wu W., Qi Z., Shen Z. (2015). Specific marker expression and cell state of schwann cells during culture in vitro. PLoS ONE.

[36] Jessen K.R., Mirsky R. (1999). Schwann cells and their precursors emerge as major regulators of nerve development. Trends Neurosci..

[37] Ao Q., Fung C.K., Tsui A.Y., Cai S., Zuo H.C., Chan Y.S., Shum D.K. (2011). The regeneration of transected sciatic nerves of adult rats using chitosan nerve conduits seeded with bone marrow stromal cell-derived schwann cells. Biomaterials.

[38] Biernaskie J., Sparling J.S., Liu J., Shannon C.P., Plemel J.R., Xie Y., Miller F.D., Tetzlaff W. (2007). Skin-derived precursors generate myelinating schwann cells that promote remyelination and functional recovery after contusion spinal cord injury. J. Neurosci..

[39] Boyd J.G., Gordon T. (2003). Glial cell line-derived neurotrophic factor and brain-derived neurotrophic factor sustain the axonal regeneration of chronically axotomized motoneurons in vivo. Exp. Neurol..

[40] Park M., Kim Y.H., Woo S.Y., Lee H.J., Yu Y., Kim H.S., Park Y.S., Jo I., Park J.W., Jung S.C. (2015). Tonsil-derived mesenchymal stem cells ameliorate ccl4-induced liver fibrosis in mice via autophagy activation. Sci. Rep..

[41] Razavi S., Ahmadi N., Kazemi M., Mardani M., Esfandiari E. (2012). Efficient transdifferentiation of human adipose-derived stem cells into schwann-like cells: A promise for treatment of demyelinating diseases. Adv. Biomed. Res..

[42] Pfaffl M.W. (2001). A new mathematical model for relative quantification in real-time RT-PCR. Nucleic Acids Res..

